# Subjective academic achievement and suicide behavior among university students in Ghana: A moderated mediation of financial stress and depression

**DOI:** 10.1371/journal.pmen.0000381

**Published:** 2026-05-29

**Authors:** Feikoab Parimah, Margaret Amenuke-Edusei, Joyce Nyarko, Collins Badu Agyemang, Ernest K. Bobo, Adina Addy, Phyllis Ama Tebuah Osei, Bernice Essien, George Ofosu Oti, Eugenia Dedo Yamson, Worlali Nyaledzigbor, Amma Serwaa Aboagye Kyei

**Affiliations:** 1 Department of Social Sciences, Central University, Accra, Ghana; 2 Department of Psychology, University of Ghana, Accra, Ghana; 3 Counselling Unit, Ghana Police Service, Accra, Ghana; PLOS: Public Library of Science, UNITED KINGDOM OF GREAT BRITAIN AND NORTHERN IRELAND

## Abstract

The study investigated the mediating role of depression in the association between subjective academic achievement and suicide behaviour. Further, we sought to find out whether financial stress would moderate such a plausible mediated relationship. A cross-sectional survey was used in the study. Out of 460 undergraduate students sampled for the study, 51.5% were male (*Mean*_*age*_ = 20.7; *SD* = 3.19). We observed that subjective academic achievement has a positive effect on depression, which in turn has a positive impact on suicide behaviour. And this relationship is not influenced by financial stress. It appears that the association between subjective academic achievement and depression is context-specific and requires further investigation, since at this stage, the relationship is fuzzy.

## Introduction

The prevalence of financial stress among university students has been reported over the past two decades. Financial stress has been noted to be a reason for contemplating suicide among premedical students in Pakistan [1]. Yang and Shim [2] found that higher financial stress increased students’ risk of suicide. Additionally, college students in financial difficulty tend to manifest depressive symptoms [3], with economic hardship leading to financial threat, which consequently leads to suicide ideation [4]. Besides, some other factors have been shown to predict suicidal behaviour among students in other parts of the world, like the United States [5]. Thus, precursors to suicidal behaviour abound, necessitating that studies within other contexts examine such precursors. The current study aims to pursue this goal by investigating how some variables, like depression and financial stress, lead to suicidal behaviour. Moreover, the relationship between depression and financial stress is also investigated to ascertain whether there will be a significant association between depression and financial stress and suicidal behaviour.

University students in some parts of Africa (e.g., South Africa) experience both financial and academic stress [6]. In Ghana, students experience academic-related stressors [7,8], including financial stress [9]. The prevalence of such stressors could lead to some psychological outcomes, such as depression, as suggested by Assari [3]. Studies on depression among college and university students have revealed a high to moderate prevalence of depression [10,11]. The presence of various levels of depression among this population is due to related risk factors such as financial challenges [12–14]. Also, the prevalence of higher depressive symptoms was reported among university students in lower and middle-income countries (LMICs) [10,14], South Africa [10,15,16], Kenya [17] and in Ghana [7] Suicide attempts have been recorded among students in Uganda [18], South Africa [16] and Ghana [19,20]. These studies merely recorded the prevalence of academic and financial stressors among university students in other parts of Africa and Ghana, failing to show the extent to which these stressors are associated with depressive symptoms among University students in Africa and Ghana. In the current study, we sought to examine the relationship between financial stress and suicidal behaviour. An examination of these associations is vital, seeing that Salifu and Yidana [19] and Owusu-Ansah et al. [20] only reported the prevalence of suicide ideation and suicide ideations, attempt, death wishes and suicidal plans, respectively, among university students in Ghana. Thus, it is not known whether factors such as financial stress and depression could have led to these.

### Academic achievement and depression

Academic achievement is the level at which students accomplish their educational ambitions, predicting students’ future success [21]. It is mostly measured using a more objective or academic performance scale, such as GPA. A significant negative relationship has been established between depression and academic success [22], academic performance [23,24], perceived academic performance [25], academic achievement [26–30], academic engagement [31], and GPA [32,33]. In a meta-analysis of longitudinal studies, Huang [34] suggested that the association between prior academic performance and subsequent depression diminishes with time. Unlike academic achievement, subjective academic achievement is more subjective, as students assess their academic successes based on their perceived effort, personal goals, and the accomplishments of their friends [35]. An incongruence between the expectations of others and how students subjectively feel about their academic performance could lead to adverse psychological outcomes. For instance, Oishi and Sullivan [36] noted that not fulfilling the expectations of one’s parents to some extent leads to lower levels of life satisfaction among American college students. Likewise, self-perception of ‘living up’ to the expectations of parents predicts psychological distress among Taiwanese college students [37]. Although a person feels s/he performed well, the expectations of significant others might have adverse psychological consequences. Many students face pressure from their families and communities to succeed academically and secure well-paying jobs. The inability to meet these expectations, or the fear of failing to do so, may contribute to deleterious psychological consequences as suggested by previous studies [36,37]. Aside from these, a positive association has been established between academic achievement and depression using objective measures [38–41]. From a Russian panel study, Dementeva and Smirnov [38] established a positive association between educational outcomes and depression for young men, and no association for young women. Similar associations have been observed in other studies with different samples [39–41]. Although the association between depression and objective measures of academic achievement is negative, some studies have established positive associations. Nevertheless, the direction of the association appears to be skewed towards the negative. Thus, it also makes sense to guess that the association between academic achievement and depression would be positive, especially when looking at subjective academic achievement. The current study seeks to examine the association between subjective academic achievement and depression.

### Academic achievement and suicide behaviour

Low academic achievement is associated with later suicide attempts [42]. Among undergraduate students in Texas, USA, suicide ideation was associated with lower GPAs [43]. Similar associations have been observed within other contexts: perceived academic performance was associated with suicidal behaviours among adolescents in South Australia [44]; self-perceived academic performance as a risk factor for suicide attempts among middle and high school students in Mexico [45]. These studies looked at objective academic achievement measures and not subjective academic achievement. So far, there is a scarcity of literature that has investigated the association between subjective academic achievement and suicidal behaviour. In the current study, we sought to examine the association between subjective academic achievement and suicidal behaviour.

### Financial stress, depression, and suicidal behaviour

Financial insecurity is likely to lead to depression among university students in a state in the United States of America [46]. Financial stress has been found to be a strong psychological stressor among Ghanaian university students [7,9]. Further, a significant positive relationship exists between depression severity and suicidal risk among university students in China [47]. Desalegn et al. [48] found that co-morbid depression symptoms and depression were significant predictors of suicide ideation and attempts, respectively, among university students in Ethiopia. In a meta-analysis, Seo et al. [49] found that depression was among the strongest predictors of suicide ideation and attempts among medical students. Hassan et al. [50] showed that financial threat predicted suicidal ideation among pre-medical students in Pakistan. Although these studies are instructive, none of them has shown the interaction effect of subjective academic achievement and financial stress on depression among university students in Ghana, and the plausible moderated mediation of financial stress and depression in the association between subjective academic achievement and suicidal behaviour among university students in Ghana. The study sought to answer two [2] research questions: [1] What is the mediating role of depression in the association between subjective academic achievement and suicidal behaviour? [2] What is the moderating role of financial stress in such a plausible mediated relationship? Consequently, we made the following conjectures:

*H*_*1*_ *a: Depression would fully mediate the association between subjective academic achievement and suicide behaviour*

*H*_*1*_ *b: Financial stress would moderate the mediated relationship*

## Materials and methods

### Research design

The study employed a cross-sectional design to examine relationships between subjective academic achievement, financial stress, depression, and suicidal behaviour among university students in Ghana. This method was chosen because it allows researchers to collect and analyse data on pre-determined variables using structured instruments such as surveys. The approach is well-suited for gathering standardised information within a single time frame.

### Study setting

The study was conducted among students of the University of Ghana and Central University. The University of Ghana is one of the public and government-funded universities in Ghana, located in Accra, in the Greater Accra Region. The University of Ghana is known to be the oldest public university with a student population of about 60,000 (about 53,000 undergraduates). The university operates a collegiate system with four [4] colleges. Each college is made up of schools/faculties. Central University is the largest private university in Ghana. The university, located in Accra, has a population of about 8, 000 undergraduates, and houses seven schools and two faculties.

### Population, sample and sampling strategy

A total of 454 students were selected through multistage sampling to participate in the study from the University of Ghana and Central University. These universities were chosen through multi-stage sampling. The multi-stage process allowed for a systematic and structured selection of participants across different stages. Universities within the Greater Accra Region of Ghana were initially clustered into public universities and private universities. It is at this stage that both the University of Ghana and Central University were drawn using simple random sampling. Further, a faculty within each of these institutions was chosen through simple random sampling. Thereafter, respondents were selected from each of the faculties of the two institutions. Undergraduate students 18 years and above were included in the study. Students below 18 years and those pursuing graduate studies were excluded from the study. The sample size was determined using Yamane’s [51] simplified formula for finite population sampling. Based on the population estimations from the two universities (University of Ghana = 66,088; Central University = 8,400), the total number of undergraduates is approximately 74,488. A 95% confidence level and a 5% margin of error yield an appropriate sample size of 398:

n = N / 1 + N(e)2

n= 74, 488 / 1 + 74, 488 (0.05)2

n = 398

### Measures

#### Financial stress scale.

The Financial Stress Scale [52] was used to assess financial stress and its manifestations in various ways. However, this study focused on one component of financial stress, specifically Affective Reaction, with a Cronbach’s alpha of 0.95. This was measured using a five-point Likert scale ranging from 1 (Strongly Disagree) to 5 (Strongly Agree), with sample items including statements such as “I worry a lot because of my financial situation” and “I feel emotionally drained because of my financial situation”

#### Depression scale.

The Patient Health Questionnaire (PHQ-9) [53] was used to measure depression. It contains nine items; each item is rated on a four-point scale (from 0 = not at all to 3 = nearly every day) (e.g., “Little interest or pleasure in doing things). The Cronbach’s alpha of the PHQ-9 ranges between  .86 and  .89.

#### Subjective Academic Achievement Scale (SAAS) scale.

The SAAS consists of five items, which participants answered on a rating scale ranging from 1 to 5 [54]. The value 1 indicated low, and the value 5 indicated high satisfaction with one’s academic achievement. Item 5 was worded negatively and thus reversed before scoring. The scale has a good internal consistency (α = .80).

#### The Suicide Behaviours Questionnaire-Revised (SBQ-R).

The SBQ-R [ 55,56] consisted of four items that measured various dimensions of suicidality: lifetime suicide ideation and suicide attempt (scored on a 4-point scale), frequency of suicidal ideation in the past 12 months (scored on a 5-point scale), the threat of suicide attempt (scored on a 3-point scale), and the likelihood of suicidal behaviour in the future (scored on a 6-point scale).

### Data analysis

Data analysis was conducted using the Statistical Package for Social Sciences (SPSS) version 24. Descriptive statistics, including frequencies and percentages, were employed to summarise the data. Pearson’s correlation analysis was utilised to assess the relationships among the study variables. Subsequently, SPSS’ PROCESS Macro model 7 was used to test the hypotheses regarding the moderated mediation analysis.

### Data collection procedure

Participants were approached at their lecture halls after class and furnished with the aims and objectives of the study. They were assured of the confidentiality of the information they gave and were informed that their information would be used solely for academic purposes. This was to help them make an informed decision regarding their participation in the study. Since all the participants were 18 years and above, written informed consent was sought and obtained from them after the study objectives had been explained to them. To keep the responses of participants anonymous, codes were used. Although none of the respondents sought psychological help, psychological help was made available for any respondent who might have needed one, during and after the interview. They would have been referred to the counselling unit at Central University or the Careers and Placement Centre at the University of Ghana if need be. This was vital since some undesirable memories were likely to be evoked in the course of the study. Apart from such possible emotional problems, there was no envisaged physical harm associated with respondents’ participation in the study. Moreover, respondents were made aware that data and results of the study will be shared with them upon request, and in case of any intervention, they will be prioritized.

### Ethics statement

We certify that all procedures performed in studies involving human participants were in accordance with the ethical standards of the institutional and/or national research committee and with the 1964 Helsinki Declaration and its later amendments or comparable ethical standards. Ethics approval was obtained from the Research Ethics Committee (UHAS-REC) (Ref: UHAS-REC A.3 [64] 23–24) of the University of Health and Allied Sciences, Ho, Ghana.

## Results

Most of the respondents were male (51.5%), with an average age of 20.75 (*SD* = 3.19). Also, a number of them were in their first year (242) and were single (432). Four hundred and thirteen (413) identified themselves as Christians (Table 1).

**Table 1:** Demographic characteristics of respondents (N = 454).

	F	%
Sex		
Male	234	51.5
Female	220	48.5
Age: M = 20.75. SD = 3.19		
Year Level		
100	242	53.3
200	73	16.1
300	59	13.0
400	50	11.0
500	30	6.6
Marital status		
Single	432	95.4
Married	11	2.4
Divorced	3	0.7
Separated	3	0.7
Cohabiting	4	0.9
Religious Affiliation		
Christianity	413	91.6
Islam	29	6.4
Traditional	4	0.9
Others	5	1.1

All the instruments were reliable (Table 2), and further analysis was conducted accordingly. Table 3 shows the bivariate correlations among the variables.

**Table 2:** Means, standard deviation, and reliability of instruments.

Instrument	N	M	SD	α
Depression	9	16.48	5.84	.78
Financial Stress	8	21.23	8.96	.94
Subjective Academic Achievement	5	14.31	3.50	.70
Suicidal Behavior	4	4.68	3.02	.83

**Table 3:** Summary descriptive analysis of the depression construct.

	Min	Max	Mean	SD	Skewness	Kurtosis
Little interest, no pleasure in doing things	1	4	1.87	.977	.924	-.188
Feeling down, depressed or hopeless	1	4	1.80	.973	.965	-.210
Trouble falling asleep, staying asleep, or sleeping too much	1	4	1.98	1.058	.762	-.682
Feeling tired or having little energy	1	4	2.14	.982	.535	-.696
Poor appetite or overeating	1	4	1.98	1.048	.743	-.695
Feeling bad about yourself or that you’re a failure or have let yourself or your family down	1	4	1.82	1.039	.991	-.329
Trouble concentrating on things such as reading the newspaper or watching television	1	4	1.84	1.000	.904	-.379
Moving or speaking slowly so that other people could have noticed. Or, the opposite of being so fidgety or restless that you have been moving around a lot more than usual	1	4	1.51	.848	1.606	1.598
Thoughts that you will be better off dead or of hurting yourself in some way	1	4	1.45	.828	1.869	2.541

### Descriptive analysis of response patterns for the study constructs

A descriptive analysis of the constructs by examining response patterns across the Likert scale for the constructs. It was noted that the response patterns for the depression construct generally clustered at the lower end (Table 3). Although the skewness and Kurtosis values fell within the acceptable range, the Shapiro-Wilk and Kolmogorov-Smirnov tests showed that the depression construct was not normally distributed (Table 4). Also, the clustering at the lower end shows that depressive symptoms among the sample were low. Unlike the depression construct, the responses on the financial stress construct were varied since most of the responses clustered around the mean or median. However, like the depression construct, the financial stress construct had skewness and kurtosis values falling within the acceptable range (Table 5), but the Kolmogorov-Smirnov and Shapiro-Wilk tests showed that the responses were not normally distributed (Table 6). Further, like the financial stress construct, responses were varied and clustered around the mean or median (Table 7). The skewness and Kurtosis values fell within the acceptable range (Table 7), but the Kolmogorov-Smirnov and Shapiro-Wilk tests showed that the data were not normally distributed (Table 8). Moreover, a floor effect was noted in the response pattern for the suicidal behaviour construct, demonstrating that respondents recorded low suicidal behaviour tendencies (Table 9). Additionally, the skewness and kurtosis values generally fell outside the range of acceptability, showing that the responses were skewed, as supported by the Kolmogorov-Smirnov and Shapiro-Wilk tests (Table 10). Owing to the failure of the constructs to meet the assumptions of normality, PROCESS Macro (Model 7) provides a non-parametric avenue to proceed with a moderated mediation analysis by making up for the violations of normality using bootstrapping.

**Table 10:** Summary of tests of normality for suicide behaviour construct.

	Kolmogorov-Smirnov^a^			Shapiro-Wilk		
	Statistic	df	Sig.	Statistic	df	Sig.
Suicide Behaviour	.312	451	<.001	.629	451	<.001

**Table 4:** Summary of tests of normality for depression construct.

	Kolmogorov-Smirnov^a^			Shapiro-Wilk		
	Statistic	df	Sig.	Statistic	df	Sig.
Depression	.115	435	<.001	.933	435	<.001

**Table 5:** Summary descriptive analysis of the financial stress construct.

	Min	Max	Mean	SD	Skewness	Kurtosis
I feel depressed because of my financial situation	1	5	2.79	1.396	.207	-1.253
I feel sad because of my financial situation	1	5	2.79	1.340	.204	-1.189
I am fearful because of my financial situation	1	5	2.53	1.252	.513	-.748
I feel anxious because of my financial situation	1	5	2.70	1.315	.286	-1.097
I worry a lot because of my financial situation	1	5	2.82	1.366	.156	-1.245
I am easily irritated because of my financial situation	1	5	2.46	1.260	.598	-.666
I feel emotionally drained because of my financial situation	1	5	2.57	1.335	.510	-.928
I feel frustrated because of my financial situation	1	5	2.67	1.370	.332	-1.153

**Table 6:** Summary of tests of normality for financial stress construct.

	Kolmogorov-Smirnov^a^			Shapiro-Wilk		
	Statistic	df	Sig.	Statistic	df	Sig.
Financial Stress	.070	448	<.001	.958	448	<.001

**Table 7:** Summary descriptive analysis of the subjective academic achievement construct.

	Min	Max	Mean	SD	Skewness	Kurtosis
I am satisfied with my grades at university	1	5	3.06	1.023	.072	-.131
I am successful in my studies	1	5	2.73	1.037	.092	-.323
My grades are appropriate for my effort	1	5	2.87	1.063	.279	-.380
I progress adequately fast in my studies	1	5	2.68	.996	.143	-.346
My fellow students study more successful than I	1	5	3.01	1.079	-.179	-.397

**Table 8:** Summary of tests of normality for subjective academic achievement construct.

	Kolmogorov-Smirnov^a^			Shapiro-Wilk		
	Statistic	df	Sig.	Statistic	df	Sig.
Subjective Academic Achievement	.077	450	<.001	.989	450	.001

**Table 9:** Summary descriptive analysis of the suicide behaviour construct.

	Min	Max	Mean	SD	Skewness	Kurtosis
Have you ever thought about or attempted to kill yourself?	1	5	1.47	.967	2.250	4.375
How often have you thought about killing yourself in the past year?	1	4	1.4368	.81779	1.808	2.216
Have you ever told someone that you were going to commit suicide, or that you might do it	1	3	1.1969	.47899	2.440	5.235
How likely is it that you will attempt suicide someday?	0	6	.59	1.273	2.572	6.392

Significant associations were observed between sex and financial stress, r(405) =-.09, *p* = .044, subjective academic achievement, r(405)=-.09, p = .034, suicide behavior, r(405), p < .01(Table 11). Also, there were significant relationships between financial stress and subjective academic achievement, r(405)=.26, *p* < .001, depression, r(405)=.46, *p* < .001, suicide behavior, r(405)=.23, *p* < .001. (Table 11). There were significant associations between subjective academic achievement and depression, r(405)=.30, *p* < .001, and suicide behavior, r(405)=.15, *p* < .01. (Table 11). Further, there was a significant association between depression and suicidal behaviour, r(405)=.31, *p* < .001 (Table 11).

**Table 11:** Bivariate correlations among variables.

	Sex	FS	SAA	DEP	TS
Sex	–	-.09**	-.09**	-.08	-.13**
FS	–	–	.26***	.46***	.23***
SAA	–	–	–	.30***	.15**
DEP	–	–	–	–	.31***
TS	–	–	–	–	–

**P < .01, *** *P < .001; Note: FS = Financial Stress; SAA = Subjective Academic Stress; DEP = Depression; TS = Total Suicidal Behaviour*

### Subjective academic achievement, depression, and suicidal behaviour

Preliminary analysis showed that the impact of sex on depression was not significant and thus was not included in the main analysis. Subjective academic achievement has a significant impact on depression (β = .44, *t* = 2.65, *p < .05*8) (Table 12). Financial stress has a significant impact on depression (β = .35, *t* = 3.20, *p* < .05). There is no in*t*eraction effect of subjective academic achievement and financial stress on depression (β = -.00, *t* = -.83, *p* = .409). Subjective academic achievemen*t* has no significant impact on suicide behaviour (β = .05, *t* = 1.25, *p* = .213). Depression has a significant impac*t* on suicide behaviour (β = .15, *t* = 6.10, *p* < .05). However, the indirec*t* effect in the presence of financial stress (at mean level) is  .05, and per the bootstrap [.02,  .09], that is within the confidence interval at a *p* < .05 (Table 12).

H_1_b suggested that the indirect effect of subjective academic achievement on suicidal behaviour through depression will be moderated by financial stress. H_1_b was not supported as the index of moderated mediation (index = -.00, 95% CI= [-.004,  .001]) was not significant since the 95% CI includes zero (Table 12).

**Table 12:** Summary of moderated mediation analysis for study constructs.

	*SAA and FS as predictors of Depression*				
R	R-sq	Adj R-sq	F	*P*	
.51	.26	.25	46.53	.000	
	*Model*				
	β	SE	t	*P*	95% CI
SAA	.44	.17	2.65	.008	[.16, .77]
FS	.35	.11	3.20	.002	[.14, .57]
	***SAA, and Depression as predictors of Suicidal Behaviour***				
R	R-sq	Adj R-sq	F	*P*	
.32	.11	.10	23.92	.000	
	*Model*				
	β	SE	t	*P*	95%CI
SAA	.05	.04	1.25	.213	[-.03, .13]
Depression	.15	.03	6.10	.000	[.10, .20]
	**Direct Effect of SAA on Suicidal Behaviour**				
	β	SE	t	P	95%CI
	.05	.04	1.25	.213	[-.03, .13]
	**Conditional indirect Effect of SAA on Suicidal Behaviour**				
*Indirect Effect*					
	FS	Effect	BootSE		95%CI
	11	.06	.02		[.03, .10]
	21	.05	.02		[.02, .09]
	31	.04	.02		[.00, .09]
	*Index of Moderated Mediation*				
	Index	BootSE			
FS	-.001	.001			[-.004, .001]

*Note: SAA = Subjective Academic Achievement; FS = Financial Stress*

## Discussion

The study sought to answer the following questions: (1) What is the mediating role of depression in the association between subjective academic achievement and suicide behaviour? (2) What is the moderating role of financial stress in such a plausible mediated relationship? It was observed that: Depression fully mediated the association between subjective academic achievement and suicide behaviour. However, financial stress failed to moderate the mediated relationship.

Depression fully mediated the association between subjective academic achievement and suicide behaviour. However, financial stress failed to moderate the mediated relationship. Previous studies have established a negative association between depression and academic achievement [26,27,29,30]. Other studies have found a negative relationship between depression and academic engagement [31], as well as academic performance [23]. Similarly, a negative association between depression and GPA [32,33], and perceived academic performance among university students in Mexico [25] have been established. It is vital to note that among adolescents in the UK, the negative association between depression and academic achievement was a bi-directional one [27]. This suggests that depression could either lead to poor academic performance or poor academic performance could lead to depression. The current study contradicts these results, in view of the positive relationship observed between subjective academic achievement and depression. Thus, the association between academic achievement and depression is not always negative. In a meta-analysis of longitudinal studies, Huang [34] suggested that the association between prior academic performance and subsequent depression diminishes with time. In essence, as the time between assessments increases, the association between academic achievement and depression decreases. Moreover, it was observed that compared to young adults, the association was stronger among children. Although several studies have established a negative association between academic achievement and depression, there are comparatively few studies that have found a positive association between academic achievement and depression. From a Russian panel study, Dementeva and Smirnov [38] established a positive association between educational outcomes and depression for young men, and no association for young women. Similarly, von Helversen et al. [41] found a positive association between depression and performance on complex sequential decision tasks among clinically depressed individuals. Such associations have been noted in Hammar and Ardal [40] and [39], who showed that performance increases with depression. Although our results align with the positive association between academic achievement and depression observed by Helversen et al. [41] and Hammar and Ardal [40], contextual nuances may help explain the findings noted in the current study. More so because subjective academic achievement was used in the current study, contrary to the objective measures used in the other studies. On one hand, within the Ghanaian cultural setting, at graduation ceremonies, a person from a village might have his or her chief come for the graduation, although the person, by an objective measure of final cumulative grade point average, would not have attained a first class, which is deemed to be high academic achievement. However, to the people from his village, that particular student (who is probably the first to have completed tertiary education) could be deemed to have performed excellently. On the other hand, a student coming from a family of scholars or high achievers may obtain, for instance, a first class, which is objectively great, but may fall short of family standards where most of the family members obtained a first class with very high cumulative grade point averages. In this case, such a student might think that though s/he has performed well, per the evaluation of significant others, that might not be good enough. In essence, the person could score high on subjective academic achievement and still show signs of depression. This could provide explanations for the positive association observed in this study.

Although financial stress predicted depression, the study found no significant interaction effect of academic achievement and financial stress on depression. Accordingly, financial stress failed to moderate the mediated relationship between subjective academic achievement and suicide behaviour, including attempt, ideation, threat, and plan. Among youths in the US, worrying about one’s economic needs has been found to negatively impact a person’s academic achievement [57]. Despite a significant negative relationship between financial stress and student academic achievement [58], Britt et al. [59] found no significant association between financial stress and academic achievement among college students in the US. The current study further underscores the significant role subjective academic achievement has on depression. As already explained, students who feel they have achieved excellently (plausibly contrary to objective standards) may still show depressive symptoms owing to the evaluations of significant others. Thus, irrespective of the financial state of the person, the evaluations of the significant others regarding his or her performance are of paramount importance.

Also, no direct association was found between subjective academic achievement and suicide behaviour. This is contrary to other studies that found a significant association between academic achievement and suicide behaviour [44]. The discrepancy between the results or the relationship observed in the current study and that of previous studies in this regard could be attributed to the measures of academic achievement. Further studies are required to firm up the association between subjective academic achievement and suicidal behaviour: attempt, ideation, threat, and plan.

### Limitations and recommendations

Despite the strengths of the study due to the sample size and sampling strategy employed, the study is not without some limitations. We acknowledge that more nuances could have been obtained to throw light on the positive association that was established between subjective academic achievement and depression, especially when it is contrary to most studies that found a negative relationship between the two. A mixed method could have made up for such a weakness since it is plausible that contextual or cultural factors could account for the association observed in the study. Future studies could use a mixed method or a qualitative study to pry into such a possible contextual nuance. Also, future studies could investigate whether the same relationship would be established if actual academic performance is used instead of the subjective academic achievement used in the current study.

## Conclusion

The study showed that subjective academic achievement does not directly impact suicide behaviour. Further, it suggests that before a student would engage in suicidal behaviour, s/he must first of all develop depressive symptoms. Thus, subjective academic performance has to necessarily lead to depression before one can engage in suicidal behaviour. And this relationship is not affected by the financial stress a student experience. This implies that school counsellors at counselling units in both public and private universities in Ghana need to watch for depressive symptoms in students and help them accordingly. By so doing, students’ subjective academic achievement would not necessarily cause them to contemplate suicide. At the beginning of each semester, students’ subjective academic achievement should be assessed alongside objective measures, such as their GPA, to identify potential discrepancies and enable subsequent intervention. This would help identify students who subjectively feel that their performance is good but could be evaluated negatively by their significant others. In such cases, familial relationships will be considered when providing counselling for students in that regard.
